# Changes in Insulin Sensitivity and Lipid Profile Markers Following Initial and Secondary Bouts of Multiple Eccentric Exercises

**DOI:** 10.3389/fphys.2022.917317

**Published:** 2022-06-06

**Authors:** Trevor C. Chen, Min-Jyue Huang, Leonardo C. R. Lima, Tai-Ying Chou, Hung-Hao Wang, Jui-Hung Tu, Shi-Che Lin, Kazunori Nosaka

**Affiliations:** ^1^ Department of Physical Education and Sport Sciences, National Taiwan Normal University, Taipei City, Taiwan; ^2^ Centre for Human Performance, School of Medical and Health Sciences, Edith Cowan University, Cowan, WA, Australia; ^3^ School of Physical Education and Sport of Ribeirão Preto, University of São Paulo, Ribeirão Preto, Brazil; ^4^ Department of Athletic Performance, National Taiwan Normal University, Taipei City, Taiwan; ^5^ Department of Physical Education, National Pingtung University, Pingtung City, Taiwan

**Keywords:** exercise-induced muscle damage, repeated bout effect, homeostasis model assessment (HOMA), plasma creatine kinase activity, total cholesterols, high-density lipoprotein cholesterols

## Abstract

An acute bout of eccentric exercise affects insulin sensitivity and lipid profile, but how the magnitude of muscle damage affects them is not clear. We compared changes in blood insulin sensitivity and lipid markers after the first (EC1) and second (EC2) eccentric exercise bouts. Fifteen sedentary young men performed arm, leg and trunk muscle eccentric exercises, and repeated them 2 weeks later. Fasting blood samples were taken before, 2 h and 1–5 days after each exercise bout to analyze plasma creatine kinase (CK) activity, serum glucose (GLU), insulin, homeostasis model assessment (HOMA), triacylglycerols (TG), total (TC) and low- (LDLC) and high-density lipoprotein cholesterol (HDLC) concentrations as well as TC/HDLC ratio. Changes in these measures were compared between bouts and relationships to peak plasma CK activity were analyzed. Plasma CK activity increased (*p* < 0.05) after EC1 (peak: 101,668 ± 58,955 IU/L) but not after EC2. The magnitude of changes in GLU (peak after EC1: 26 ± 10% vs. EC2: 7 ± 6%), insulin (46 ± 27% vs. 15 ± 8%), HOMA (86 ± 48% vs. 24 ± 15%), TC (−20 ± 5% vs. −6 ± 4%), TG (−32 ± 11% vs. −6 ± 3%), LDHC (−47 ± 15% vs. −12 ± 9%), HDLC (35 ± 26% vs. 7 ± 4%), and TC/HDLC ratio (−139 ± 13% vs. −11 ± 7%) were significantly greater after EC1 than EC2. Peak plasma CK activity was significantly (*p* < 0.05) correlated with the peak changes in blood insulin sensitivity and lipid markers for the combined data of EC1 and EC2. These results suggest that the greater the magnitude of muscle damage, the greater the magnitude of changes in the insulin sensitivity to a negative direction and lipid markers to a positive direction.

## Introduction

Resistance exercise training induces not only muscle adaptations such as increases in muscle strength and mass, but also positive effects on insulin sensitivity and blood lipid profile (Tsekouras et al., 2009; Bacchi et al., 2012). Generally, chronic adaptations to an exercise training can be predicted by acute responses to a bout of exercise (Thompson et al., 2001). However, changes in insulin sensitivity and blood lipid profile markers after an acute resistance exercise are not necessarily reflective of those after resistance exercise training. For example, Gordon et al. (2012) reported that resting serum glucose concentration (∼176%) and insulin area under the curve (∼82%) increased for 4 days following resistance exercise consisting of three sets of 10 repetitions of bench press, leg press, shoulder press, calf raises, and lateral pull-down performed by healthy untrained older adults, which indicated a decrease in insulin sensitivity. Regarding lipid metabolism markers, Tsekouras et al. (2009) reported that plasma triacylglycerol (TG) concentration reduced (-21%) immediately after resistance exercises consisting of leg press, leg pull, knee flexion, knee extension, shoulder press, chin ups, chest press, upright row, hip flexion, hip extension, shoulder abduction, and shoulder adduction performed by healthy recreational active young men. This suggests some positive effects on lipid metabolism even after an acute resistance exercise bout.

Lee et al. (2020) reported that an acute bout of 50 maximal isokinetic (20^o^/s) eccentric contractions of the trunk extensors significantly increased resting serum glucose concentration by more than 10% and decreased TG by more than 11% at 2–3 days post-exercise, but no such effect was observed after concentric contractions that did not induce muscle damage. Thus, it is necessary to understand the effects of muscle damage on insulin sensitivity and blood lipid profile. In fact, Asp et al. (1995) suggested that insulin resistance and impaired muscle glycogen resynthesis after eccentric exercise were associated with muscle damage that resulted in a decrease in glucose transporter 4 (GLUT-4) protein concentration in the muscle. Eccentric contractions could mechanically disrupt muscle plasma membrane and t-tubule network (Cooper and McNeil, 2015), which could affect transmembrane substrate transporters such as GLUT-4. Sidky et al. (2022) recently reported that dystrophin (+136%), β-sarcoglycan (+56%), and junctophilin (+58%) in tibialis anterior and extensor digitorum longus of mice increased after six bouts of 50 maximal eccentric contractions *in vivo* performed every 7 days between bouts. It is possible that membrane integrity is strengthened even after the initial eccentric exercise bout, reducing the negative effects of eccentric exercise-induced muscle damage on glucose transporters and intracellular glucose disposal. It is well known that the magnitude of muscle damage is substantially attenuated when the same eccentric exercise is repeated within several weeks, which is known as the repeated bout effect (Hirose et al., 2004; Smith et al., 2007; Hubal et al., 2008; Hyldahl et al., 2017). Thus, it is important to compare the first and second eccentric exercise bouts for acute changes in insulin sensitivity and lipid profile markers, in order to better understand the acute effects of eccentric exercise on glucose and lipid metabolism.

Considering the fact that resistance exercises of several different muscle groups are commonly included in exercise sessions performed in a gym, it is interesting to investigate how multiple resistance exercises focusing on eccentric contractions affect insulin sensitivity and lipid profile markers. It is also possible that we could magnify the effects of muscle damage on insulin sensitivity and lipid profile markers by investing an exercise in which multiple muscles are involved, potentially affecting more muscles. Thus, we used blood samples stored in a deep freezer from a previous study that compared between the first and second bouts of arm, leg and trunk muscle eccentric exercises separated by 2 weeks for changes in indirect muscle damage markers such as maximal voluntary isometric contraction (MVC) strength, delayed onset of muscle soreness (DOMS), plasma CK activity and myoglobin concentration (Chen et al., 2019b). It is important to note that the study was unique, because peak plasma CK activity after the first bout was 101,668 ± 58,955 (range: 23,238–207,304 IU/L), which was very high, but no significant increase was found after the second bout (Chen et al., 2019b). Although CK activity in the blood is not a reliable marker of muscle damage as some studies reported (Gunst et al., 1998; Paulsen et al., 2012), large increases in plasma or serum CK activity (e.g., >10,000 IU/L) reflect severe muscle membrane damage (Clarkson et al., 2006; Paulsen et al., 2012). Thus, it seems likely that muscle membrane damage was severe after the first whole-body eccentric exercises, but little after the second bout. This provided us a better opportunity to examine the effects of muscle membrane damage on insulin sensitivity or lipid profile markers.

Therefore, the purpose of the present study was to investigate the hypothesis that changes in insulin sensitivity and lipid markers after the first bout of whole-body eccentric exercise would be greater than those after the second bout of the same exercise performed 2 weeks later. The present study measured serum glucose, insulin, homeostasis model assessment (HOMA), TG, total (TC) and low- (LDLC) and high-density lipoprotein cholesterols (HDLC) concentrations and TC/HDLC ratio to investigate changes in these measures following the first and second eccentric exercise bouts and their relationships to peak plasma CK activity.

## Materials and Methods

### Participants and Study Design

The details of the study except for the blood measures specific to the present study can be found in the previous study (Chen et al., 2019b). Briefly, 15 sedentary young men (mean ± SD age: 21.5 ± 1.6 years, height: 173.2 ± 5.2 cm, body mass: 72.6 ± 15.6 kg) were recruited, and they provided informed consents to participate in the study that had been approved by the Institute of Research Ethics Committee. The study was conducted in conformity with the policy statement regarding the use of human subjects by the Declaration of Helsinki.

The participants performed two bouts of nine eccentric exercises (EC1 and EC2) consisting of arm curl (target muscles: elbow flexors), arm extension (elbow extensors), chest press (pectoralis), leg extension (knee extensors), leg curl (knee flexors), standing calf raise (plantar flexors), lat pulldown (latissimus), abdominal crunch (abdominis) and back extension (erector spinae) on nine different resistance training machines (Cybex International, Inc., Owatonna, MN., United States), with 2 weeks between bouts (Chen et al., 2019b). For each exercise, the load was set at 80% of MVC strength, and 5 sets of 10 eccentric contractions with a 15-s rest between contractions and a 2-min rest between sets, were performed. In order to minimize neuromuscular fatigue by concentric contractions, the concentric phase was assisted by one or two researchers. Each eccentric contraction lasted for 5 s as guided by the researcher who counted “0, 1, 2, 3, 4, 5” for the movement. The participants resisted the load from a short muscle length (i.e., the starting angle) to a long muscle length (i.e., the finishing angle), and after each contraction at the end of the range of motion, the researcher reset the machine to the starting position while the participants were relaxing.

### Blood Analyses

The blood analyses consisted of plasma CK activity, serum glucose, insulin, HOMA, TG, TC, LDLC, HDLC concentrations as well as TC/HDLC ratio. The blood samples were collected after 10-hour of fasting between 7:00–9:00 a.m. except for the time point of 2-hour post-exercise. Blood sample was drawn by a standard venipuncture technique from the cubital fossa region of the arm to two 5-ml tubes; one containing ethylenediaminetetraacetic acid (Becton Dickinson and Company, Plymouth, United Kingdom) and another serum separation tube. The blood was clotted at room temperature for the serum sample, and the tubes were centrifuged at 3,000 rpm for 10-minute to obtain plasma and serum, respectively, and the samples were separated to several tubes and stored at −80°C until analyses.

Plasma CK activity was analyzed spectrophotometrically by an automated clinical chemistry analyzer (Model 7080; Hitachi, Co. Ltd., Tokyo, Japan) using a commercially available test kit (e.g., Chou et al., 2018; Chen et al., 2019a, Chen et al., 2019b; Kang et al., 2022). Serum glucose concentration was assayed by a Beckman Unicel DxC 600/800 Chemistry Analyzer (Beckman Coulter Inc., Fullerton, CA, United States of America) using a commercially available kit (GLUCm) (e.g., Smith et al., 2020; Tsai et al., 2021). Serum insulin concentration was analyzed by an immunoradiometric assay kit (INS-IRMA kit; Biosource, Nivelles, Belgium) using a gamma counter system (MIC Group, Inc., Ramsey, MN, United States of America) (e.g., Chen et al., 2017a; Lee et al., 2018). HOMA was calculated as fasting insulin (μU/mL) fasting glucose (mmol/L)/22.5 (Paschalis et al., 2011; Chen et al., 2017a). The normal reference ranges for glucose and insulin were 3.9–5.6 mmol/L and 28.6–114.3 pmol/L, respectively, based on the manufacturer’s information.

Blood lipid profile parameters consisted of serum TG, TC, HDLC, LDLC concentrations, and TC/LDHC ratio (e.g., Paschalis et al., 2011; Kuo et al., 2020; Cheng et al., 2022). These measures were performed by a Beckman Unicel DxC 600/800 Chemistry Analyzer using commercial kits (Beckman Coulter, Inc., Brea, CA, United States), and TC/HDLC ratio (considered an atherogenic index) was calculated (Paschalis et al., 2011). The reference ranges of TG, TC, HDLC, and LDLC was <1.6 mmol/L, <5.2 mmol/L, ≥1.6 mmol/L, and <2.6 mmol/L, respectively, based on the manufacturer’s information.

The test–retest reliability of each measure was determined by coefficient of variation (CV) can be found in the previous studies (Chen et al., 2017a; Chen et al., 2017b; Chen et al., 2021). The CV of plasma CK activity, serum glucose, insulin, HOMA, TG, TC, HDLC and LDLC concentrations as well as TC/HDLC ratio was 8.0%, 5.3%, 0.4%, 7.1%, 3.7%, 1.8%, 7.0%, and 1.8% as well as 3.7%, respectively.

### Statistical Analyses

Baseline values of the blood measures before EC1 and EC2 were compared by paired t-tests. Changes in plasma CK activity, and serum glucose, insulin, HOMA, TG, TC, HDLC and LDLC concentrations as well as TC/LDHC ratio over time were compared between EC1 and EC2 by a two-way repeated-measures analysis of variance (ANOVA). When a significant interaction effect was found, a Tukey’s post-hoc test was performed. Eta-squared values (η^2^) were calculated as measures of effect size, and they were considered ∼0.02 as small effect, ∼0.13 as medium effect and >0.26 as large effect (Bakeman, 2005). Additionally, one-way repeated-measure ANOVA was used to examine changes in the blood measures before, 2 h, and 1–5 days following each exercise bout. Pearson product-moment correlation coefficients were used to assess the relationships between peak plasma CK activity, and peak changes in serum glucose, insulin, HOMA, TG, TC, HDLC and LDLC concentrations as well as TC/LDHC ratio after EC1 (*n* = 15) and EC2 (*n* = 15) separately, and EC1 and EC2 by pooling them together (N = 30). A significance level was set at *p* ≤ 0.05 for all analyses. The data were presented as mean ± standard deviation (SD).

## Results

### Baseline Measurements

**FIGURE 1:**
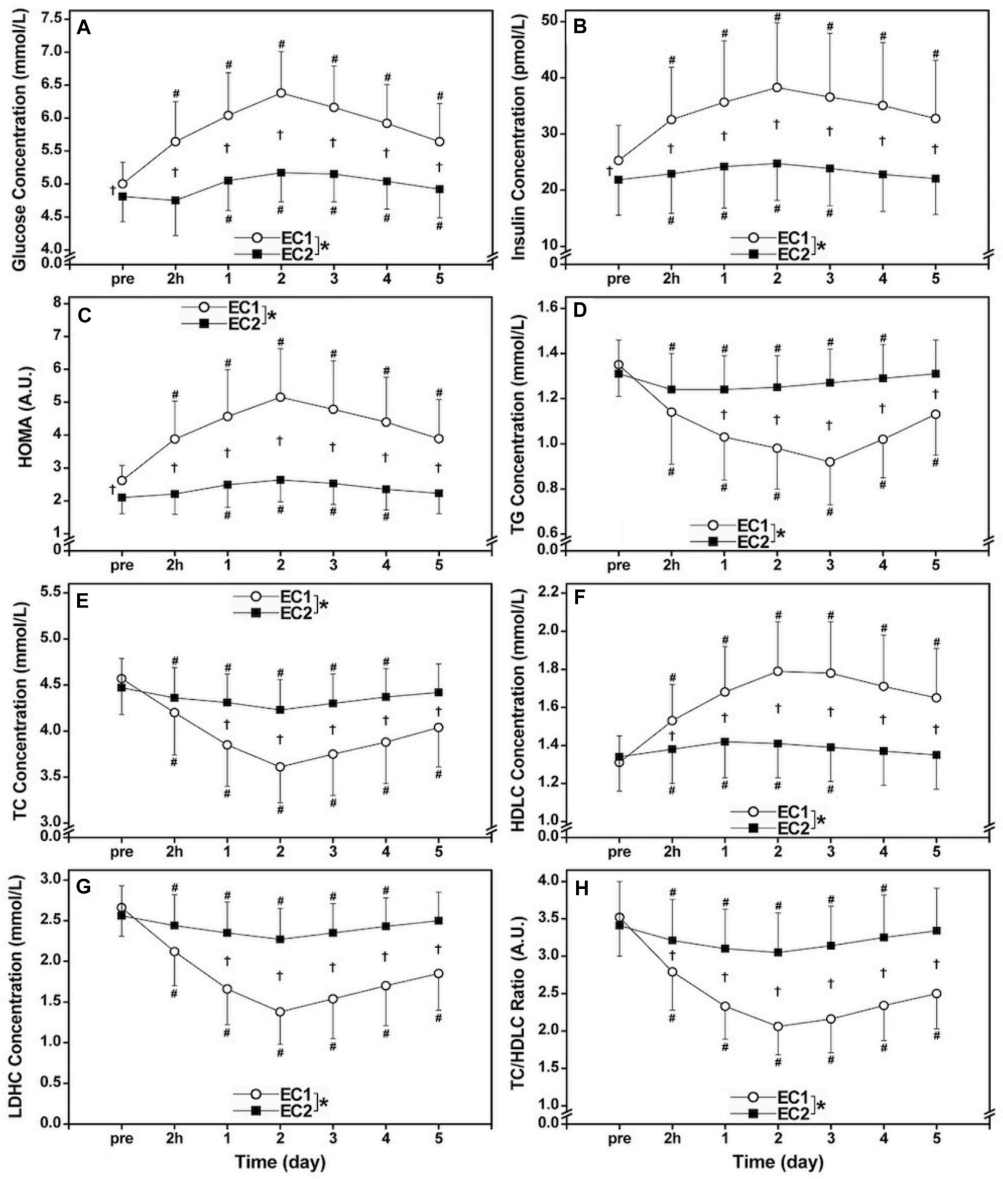
Changes (mean ± SD) in serum glucose **(A)**, insulin **(B)**, and homeostasis model assessment (HOMA, **(C)**, triacylglycerols [TG, **(D)**], total [TC, **(E)**] and high- [HDLC, **(F)**] and low-density lipoprotein cholesterols [LDLC, **(G)**] concentrations as well as TC/HDLC ratio **(H)** before (pre) and 2 h (2 h), 1, 2, 3, 4, and 5 days (1–5) after the first bout (EC1) and second bout (EC2) of whole-body eccentric exercises. *: a significant (*p* < 0.05) difference between EC1 and EC2 based on the interaction effect shown by the ANOVA. ^†^: a significant (*p* < 0.05) difference between bouts for each time point based on the post hoc test. ^#^: a significant (*p* < 0.05) difference from the baseline.

No significant differences (*p* > 0.05) in the baseline values of serum TG, TC, HDLC and LDHC concentrations as well as TC/HDLC ratio were evident between bouts (Figures 1D–H). However, serum glucose (4.81 ± 0.38 mmol/L) and insulin (21.8 ± 6.3 pmol/L) concentrations as well as HOMA (2.10 ± 0.49) before EC2 were significantly (*p* < 0.05) smaller than those before EC1 (glucose: 5.00 ± 0.33 mmol/L, insulin: 25.2 ± 6.3 pmol/L, HOMA: 2.64 ± 0.46) (Figures 1A–C).

### Changes in Insulin Sensitivity and Lipid Profile Markers After Exercise

Figure 1 compared between EC1 and EC2 for changes in serum glucose, insulin, HOMA, TG, TC, HDLC and LDLC concentrations as well as TC/LDHC ratio. Serum glucose (EC1: η^2^ = 0.787, EC2: η^2^ = 0.686), insulin (EC1: η^2^ = 0.693, EC2: η^2^ = 0.535), HOMA (EC1: η^2^ = 0.768, EC2: η^2^ = 0.712), TG (EC1: η^2^ = 0.767, EC2: η^2^ = 0.715), TC (EC1: η^2^ = 0.831, EC2: η^2^ = 0.479), HDLC (EC1: η^2^ = 0.689, EC2: η^2^ = 0.595) and LDHC (EC1: η^2^ = 0.818, EC2: η^2^ = 0.513) concentrations, and TC/HDLC ratio (EC1: η^2^ = 0.788, EC2: η^2^ = 0.597) changed significantly (*p* < 0.001) following both bouts (Figure 1). However, the changes in glucose (interaction effectη^2^ = 0.663), insulin (η^2^ = 0.639), HOMA (η^2^ = 0.754), TG (η^2^ = 0.762), TC (η^2^ = 0.669), HDLC (η^2^ = 0.663), LDHC (η^2^ = 0.780), and TC/HDLC ratio (η^2^ = 0.708) were significantly (*p* < 0.001) smaller after EC2 than EC1.

### Correlations Between Changes in Peak CK and Other Measures

**FIGURE 2:**
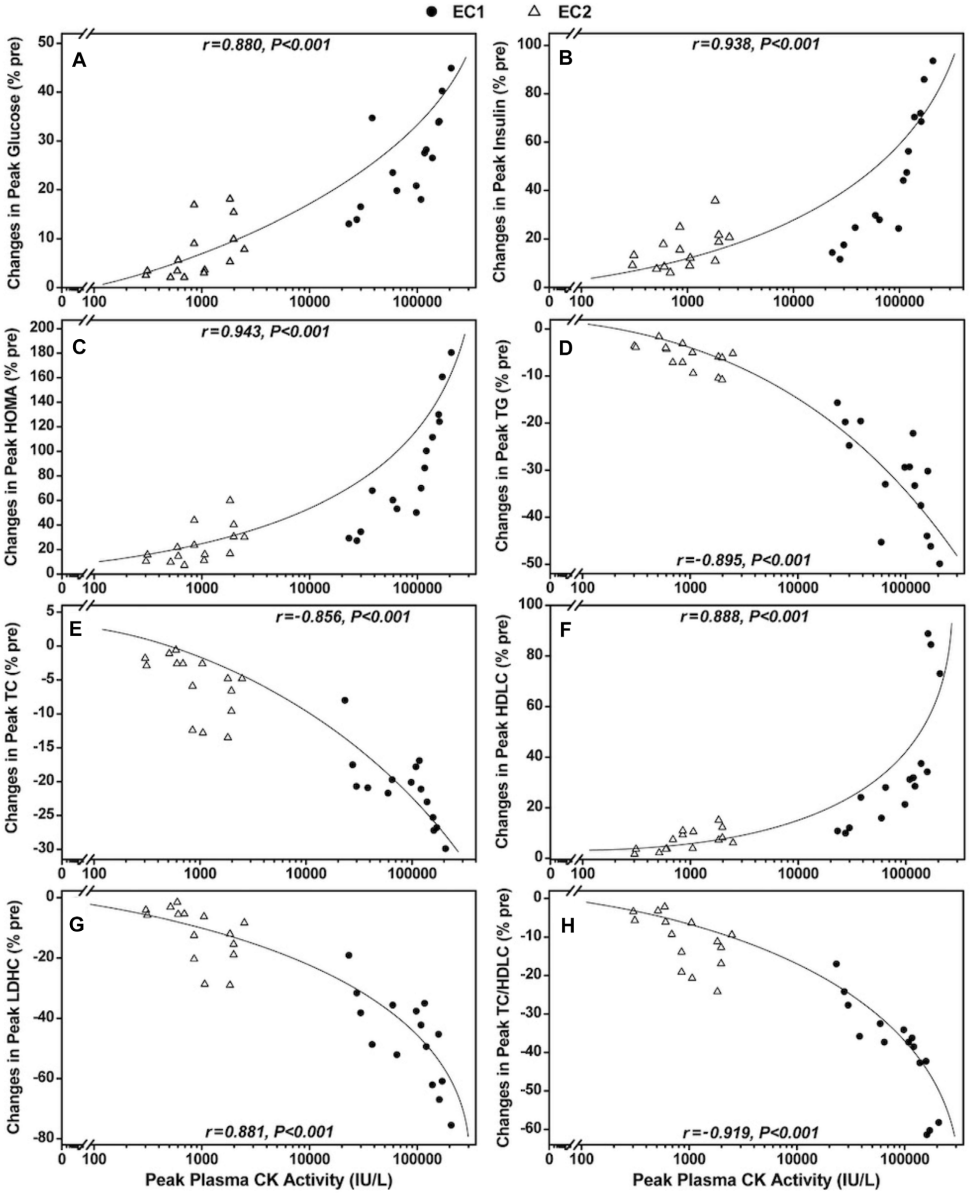
Correlations between peak plasma creatine kinase (CK) activity and peak changes in serum glucose **(A)**, insulin **(B)**, homeostasis model assessment [HOMA, **(C)**], triacylglycerols [TG, **(D)**], total [TC, **(E)**] and low- [LDLC, **(F)**] and high-density lipoprotein cholesterols [HDLC, **(G)**] concentrations as well as TC/HDLC ratio **(H)** and following the first (●) and second (∆) bouts of whole-body eccentric exercises (N = 30).

Figure 2 shows the relationships between peak plasma CK activity and the peak changes in serum glucose, insulin, HOMA, TC, TG, HDLC and LDLC concentrations as well as TC/HDLC ratio. When focusing on EC1 only (*n* = 15), a significant (*p* < 0.01) correlation was found between the peak plasma CK activity and peak glucose (*r* = 0.793), insulin (*r* = 0.965), HOMA (*r* = 0.944), TC (*r* = −0.755), TG (*r* = −0.723), HDLC (*r* = 0.830) and LDLC (*r* = −0.778) concentrations as well as TC/HDLC ratio (*r* = −0.882). As for EC2 only (*n* = 15), a significant (*p* < 0.05) correlation was also observed between the peak plasma CK activity and peak glucose (*r* = 0.530), insulin (*r* = 0.540), HOMA (*r* = 0.569), TG (*r* = −0.538), HDLC concentrations (*r* = 0.555), but no significant correction was found between the peak plasma CK activity and peak TC (*r* = −0.451, *p* = 0.092) and LDLC concentrations (*r* = −0.472, *p* = 0.076) as well as TC/HDLC ratio (*r* = −0.501, *p* = 0.057). When pooling the data from EC1 and EC2 (N = 30), significant (*p* < 0.001) correlations were still evident between the peak plasma CK activity and peak glucose (*r* = 0.880), insulin (*r* = 0.938), HOMA (*r* = 0.943), TG (*r* = −0.895), TC (*r* = −0.856), HDLC (*r* = 0.888) and LDHC (*r* = −0.881) concentrations, as well as TC/HDLC ratio (*r* = −0.919) after EC1 and EC2.

## Discussion

The main findings of the present study were follows. 1) Changes in all insulin sensitivity (glucose and insulin concentrations, HOMA) and lipid markers (TG, TC, HDLC and LDHC concentrations, TC/HDLC ratio) were significantly greater following EC1 than EC2, although the changes after EC2 were still significant (Figure 1). 2) The peak plasma CK activity significantly correlated with the peak changes in insulin sensitivity and lipid markers (Figure 2). These results supported the hypothesis that changes in the insulin sensitivity and lipid markers would be greater after EC1 than EC2.

### Insulin Sensitivity

The magnitude of peak increases in glucose (26%), insulin (46%) and HOMA (86%) after EC1 was significantly greater than that after EC2 (7%, 15%, and 24%, respectively) as shown in Figures 1A–C. Green et al. (2010) reported significant increases in insulin (38%) and glucose area under the curve (21%) at 2 days after the first bout of 30-min downhill running, but not after the second bout that was performed 2 weeks later. They speculated that the increases in insulin and glucose after the first exercise bout were due to a failure to adequately dispose blood glucose because of muscle damage. It should be noted that the magnitude of increases in the glucose, insulin and HOMA after EC1 in the present study was greater than that after an acute bout of bilateral knee flexor eccentric exercise (glucose: 6%, insulin: 18%, HOMA: 22%) (Paschalis et al., 2011) or trunk extensor eccentric exercise (glucose: 12%, insulin: 18%, HOMA: 22%) (Lee et al., 2020). The greater increases in glucose, insulin and HOMA in the present may be associated with the greater muscle damage indicated by the much greater increases in plasma CK activity (peak: 101,668 IU/L), when compared with those in the previous studies (<3,000 IU/L) (Paschalis et al., 2011; Lee et al., 2020). It is likely that more muscles and muscle plasma membrane were affected by the whole-body exercises, which reduced the disposal of glucose to skeletal muscles greater. It is important to note that increases in plasma glucose and insulin concentrations and HOMA increased after EC2. If these changes are considered to represent a decreased insulin sensitivity, the insulin sensitivity was decreased acutely after EC2, although its extent was much smaller than after EC1.

As shown in Figure 2, significant correlations between peak plasma CK activity and insulin sensitivity markers (glucose, insulin, HOMA) were also found. This suggests that the greater magnitude of the muscle membrane damage (Brancaccio et al., 2007), the greater the magnitude of increase in the insulin resistance. It is possible that muscle membrane damage affects transmembrane substrate transporters (Cooper and McNeil, 2015). Asp et al. (1995) reported that significant increases in blood glucose level at 1 day (+14%) after eccentric cycling exercise of one-leg, together with significant decreases in muscle concentration of the glucose transporter GLUT-4 protein (−44%–−46%) and muscle glycogen concentrations (−11%–−17%) at 1–2 days and immediately to 2 days post-exercise, respectively. They stated that insulin resistance and impaired muscle glycogen resynthesis after eccentric exercise were associated with a decrease in GLUT-4 protein concentration in the muscle. It is possible that the greater decrease in insulin sensitivity (or the increases in insulin resistance) after EC1 than EC2 was due to impaired glucose transport into muscle cells associated with decreases in GLUT-4 concentrations in the muscles after eccentric exercise. Aoi et al. (2013) stated that the decreased glucose utilization was caused by decreased insulin-stimulated glucose uptake in damaged muscles with inhibition of the membrane translocation of GLUT-4, and inflammatory cytokines, reactive oxygen species including 4-hydroxy-2-nonenal and peroxynitrate can induce degradation or inactivation of signaling proteins through posttranslational modification, thereby resulting in a disturbance in insulin signal transduction. Sidky et al. (2022) showed increases in dystrophin, β-sarcoglycan, and junctophilin in mice hindlimb muscles after repeated bouts of 50 maximal eccentric contractions, and concluded that the increased dystrophin, β-sarcoglycan, and junctophilin reduced eccentric contraction-induced membrane strain. If this is also the case for human, it seems possible that maintenance of membrane integrity following EC2 reduced the disruptions of insulin receptor and maintained functions of glucose transporters and intracellular glucose disposal.

### Lipid Profile

Changes in TC (−20%), TG (−32%), HDLC (35%), LDHC (−47%), TC/HDLC ratio (−39%) following EC1 were significantly greater than those after EC2 (−6%, −6%, 7%, −12%, and −11%, respectively) as shown in Figures 1D–H. These results were in line with the findings by Nikolaidis et al. (2008) who showed that the magnitude of changes in TC (peak: −14%), TG (−18%), LDHC (−25%) and TC/HDLC ratio (−20%) after EC1 of the unilateral knee flexors were significantly greater than that after EC2 (−10%, −8%, −18%, and −15%, respectively), without difference in HDLC between bouts (7–8%). The authors speculated that the decrease in serum TG concentration after EC1 was associated with the increased activity of lipoprotein lipase (LPL) that acted on lipoprotein particles passing through the capillaries, releasing free fatty acids that could be taken up by skeletal muscle and either esterified in phospholipids and intramuscular TG or oxidized in the mitochondria (Nikolaidis et al., 2008). Increased LPL activity is related to the increased demand of the working muscle for fatty acids as energy-yielding substrate and to the replenishment of muscle phospholipid and TG stores with fatty acids for the regeneration of damaged muscle fibers (Jansson and Kaijser, 1987; Oscai et al., 1990). Moreover, the decreased serum TG levels after EC1 may be due to the higher levels of resting energy expenditure that lasts for several days after eccentric exercise (Dolezal et al., 2000; Hackney et al., 2008), where there is increased need for adenosine triphosphate mainly for the regeneration of damaged and/or for the formation of new muscle fibers from satellite cells.

Nikolaidis et al. (2007) reported that glutathione (GSH: −40% vs. −19%) and GSH/oxidized glutathione (GSSG: –63% vs. –43%) decreased greater after the first than second bout of eccentric exercise of the knee flexors, whereas GSSG (+54% vs. +33%), thiobarbituric acid–reactive substances (+73% vs. +25%), protein carbonyls (+82% vs. +32%), catalase (+70% vs. +22%), uric acid, (+15% vs. +4%) bilirubin (+21% vs. +8%), and total antioxidant capacity (+48% vs. +17%) increased less after the first than the second bout. These showed that oxidative stress was greater after EC1 than EC2. Nikolaidis et al. (2008) speculated that the more favorable changes in lipid profile after the first than second eccentric exercise were related to the greater oxidative stress after the first exercise bout. Thus, muscle damage appears to induce favorable effects on blood lipid profile temporarily.

As shown in Figures 2D–H, significant correlations between peak CK activity and all lipid profile markers were found. The greater the peak CK activity, the greater the changes in lipid profiles to a better direction. Since cholesterol constitutes approximately 13% of muscle membranes (Guyton, 1981), it may be that the decreases in serum TC and LDLC concentrations after eccentric exercise were associated with outflow of cholesterol from plasma in muscle-promoting synthesis of new cell membranes (Nikolaidis et al., 2008; Paschalis et al., 2010). Thus, the depressed serum TC and LDLC concentrations observed following EC2 (Figures 1E,F) may be, at least in part, due to the less muscle damage because less cholesterol molecules would be needed for the repair process that takes place in the damaged muscle cells. It appears that muscle damage is favorable for changes in blood lipid profile acutely, but it should be noted that the blood lipid profile changes to a favorable direction even after eccentric exercise without muscle damage as shown in the second eccentric exercise bout (no significant increase in plasma CK activity). Thus, it does not mean that muscle damage is good to improve blood lipid profile.

### Practical Application

It is interesting that the effects of muscle damage on the lipid markers were the direction of favorable responses, in contrast to the glucose markers that showed unfavorable responses. It should be noted that the favorable or unfavorable changes after eccentric exercise found in the present study were acute responses, and the variables of both insulin sensitivity and lipid profile markers returned to the pre-exercise levels in 2 weeks before performing EC2 (Figure 1). Thus, even if muscle damage produces favorable responses of blood lipid profile, it does not necessarily mean that it is better to damage muscles to improve blood lipid profile. Although the changes were smaller, EC1 changed the lipid markers in favorable direction, and importantly, changes in the insulin sensitivity markers were much smaller after EC2 (Figure 1).

Several studies have shown that eccentric exercise training without muscle damage improved insulin sensitivity and blood lipid profile (Chen et al., 2017a; Chen et al., 2017b; Drexel et al., 2021). For example, Chen et al. (2017b) reported that progressive eccentric resistance training of the knee extensors over 12 weeks decreased resting levels of insulin, glucose, HOMA and OGTT, and decreased TG and TC. The improved insulin sensitivity after chronic eccentric training may be related to the increased fat oxidation (Paschalis et al., 2011). Since eccentric resistance exercise produces less cardiovascular stress (Meyer et al., 2003) and less fatigue (Horstmann et al., 2001) than concentric resistance exercise, eccentric resistance exercise may be a more suitable exercise modality for people with impaired endurance such as patients with chronic diseases. It is interesting to investigate a long-term effect of the whole-body eccentric exercises on insulin sensitivity and lipid profile.

### Limitations of the Study

There were several limitations in the present study. Firstly, the present study used relatively smaller number of young health sedentary men, thus the findings of the present study could not be generalized to other populations. Secondly, body composition, daily food intake and physical activity levels were not monitored in the present study. It is possible that these factors also affected the outcome measures. Thirdly, the present study only analyzed blood insulin sensitivity and lipid profile markers in relation to plasma CK activity as a muscle damage marker. It is interesting to investigate the relationships between other markers of muscle damage such as muscle function and muscle soreness and blood insulin sensitivity and lipid profile markers. However, it appears that plasma CK activity represent muscle plasma membrane damage well. Muscle biopsy technique to examine histological changes in muscle fibers and measuring energy markers such as adiponectin are necessary to understand the mechanisms underpinning the effects of muscle damage on glucose and lipid metabolism. Fourthly, there was no control or comparison group (e.g., concentric exercise) in the present study. Thus, it is not known whether the changes in the blood insulin sensitivity and lipid profile markers were peculiar to eccentric exercise, and how whole-body concentric exercises affect the markers. Lastly, it is not known whether the whole-body eccentric exercises are more effective than other eccentric exercises, when they are performed regularly for a longer period of time (i.e., training), to improve insulin sensitivity and lipid profile.

## Conclusion

The present study showed the greater the magnitude of muscle damage induced by eccentric exercise, the more negative effects on the insulin sensitivity markers but the more favorable effects on the blood lipid profile markers. Further studies are warranted to investigate how muscle damage affected them, the mechanisms underpinning the repeated bout effect on the insulin sensitivity and lipid markers, and the effects of whole-body eccentric exercise training on obesity, metabolic syndrome, type 2 diabetes mellitus and other metabolic diseases.

## Data Availability

The raw data supporting the conclusion of this article will be made available by the authors, without undue reservation.
